# Identification of eight novel proteasome variants in five unrelated cases of proteasome-associated autoinflammatory syndromes (PRAAS)

**DOI:** 10.3389/fimmu.2023.1190104

**Published:** 2023-08-04

**Authors:** Jonas Johannes Papendorf, Frédéric Ebstein, Sara Alehashemi, Daniela Gerent Petry Piotto, Anna Kozlova, Maria Teresa Terreri, Anna Shcherbina, Andre Rastegar, Marta Rodrigues, Renan Pereira, Sophia Park, Bin Lin, Kat Uss, Sophie Möller, Ana Flávia da Silva Pina, Flavio Sztajnbok, Sofia Torreggiani, Julie Niemela, Jennifer Stoddard, Sergio D. Rosenzweig, Andrew J. Oler, Colton McNinch, Marietta M. de Guzman, Adriana Fonseca, Nicole Micheloni, Melissa Mariti Fraga, Sandro Félix Perazzio, Raphaela Goldbach-Mansky, Adriana A. de Jesus, Elke Krüger

**Affiliations:** ^1^ Institut für Medizinische Biochemie und Molekularbiologie (IMBM), Universitätsmedizin Greifswald, Greifswald, Germany; ^2^ Translational Autoinflammatory Diseases Section (TADS), Laboratory of Clinical Immunology and Microbiology, National Institute of Allergy and Infectious Diseases, National Institutes of Health, Bethesda, MD, United States; ^3^ Division of Pediatric Rheumatology, Department of Pediatrics, Universidade Federal de São Paulo (Unifesp), São Paulo, Brazil; ^4^ Department of Immunology, D.Rogachev National Medical and Research Center for Pediatric Hematology, Oncology, and Immunology, Moscow, Russia; ^5^ Division of Pediatric Rheumatology, Department of Pediatrics, Universidade Federal do Rio de Janeiro (UFRJ), Rio de Janeiro, Brazil; ^6^ Department of Pediatrics, Universidade Federal de Ciencias da Saude de Porto Alegre, Porto Alegre, Brazil; ^7^ Immunology Service, Department of Laboratory Medicine, Clinical Center, National Institutes of Health, Bethesda, MD, United States; ^8^ Bioinformatics and Computational Biosciences Branch, Office of Cyber Infrastructure and Computational Biology, National Institute of Allergy and Infectious Diseases, National Institutes of Health, Bethesda, MD, United States; ^9^ Section of Pediatric Rheumatology, Texas Children’s Hospital, Baylor College of Medicine, Houston, TX, United States; ^10^ Division of Rheumatology – Department of Medicine, Universidade Federal de São Paulo (Unifesp), Sao Paulo, Brazil

**Keywords:** proteasomopathy, proteasome associated autoinflammatory syndrome, type I interferon, interferonopathy, PSMB8, PSMB10, PSMA5, PSMC5

## Abstract

Mutations in genes coding for proteasome subunits and/or proteasome assembly helpers typically cause recurring autoinflammation referred to as chronic atypical neutrophilic dermatosis with lipodystrophy and elevated temperatures (CANDLE) or proteasome-associated autoinflammatory syndrome (PRAAS). Patients with CANDLE/PRAAS present with mostly chronically elevated type I interferon scores that emerge as a consequence of increased proteotoxic stress by mechanisms that are not fully understood. Here, we report on five unrelated patients with CANDLE/PRAAS carrying novel inherited proteasome missense and/or nonsense variants. Four patients were compound heterozygous for novel pathogenic variants in the known CANDLE/PRAAS associated genes, *PSMB8* and *PSMB10*, whereas one patient showed additive loss-of-function mutations in *PSMB8*. Variants in two previously not associated proteasome genes, *PSMA5* and *PSMC5*, were found in a patient who also carried the *PSMB8* founder mutation, p.T75M. All newly identified mutations substantially impact the steady-state expression of the affected proteasome subunits and/or their incorporation into mature 26S proteasomes. Our observations expand the spectrum of PRAAS-associated genetic variants and improve a molecular diagnosis and genetic counseling of patients with sterile autoinflammation.

## Introduction

Protein breakdown is ensured by two main cellular degradation machineries: the ubiquitin-proteasome system (UPS) and the autophagy lysosomal system (1). The UPS is a constitutively active process (2) involved in the rapid elimination of a large variety of protein substrates, which are typically modified with ubiquitin chains and are subsequently degraded by the 26S proteasomes into shorter peptides (3). Proteasomal substrates include newly synthetized, misfolded, and/or damaged proteins that may arise during translation to mature full-length proteins in response to specific stimuli. The conjugation of protein substrates with ubiquitin moieties requires the coordinated action of the E1 ubiquitin-activating enzyme, the E2 ubiquitin-conjugating enzymes, and the E3 ubiquitin ligases (4). At least 50 genes encoding proteasome subunits and proteasome assembly chaperones contribute to the formation of different proteasome complexes in cells (5). The 26S proteasome is a multiprotein complex consisting of a 20S core particle containing the active sites and a 19S regulatory particle, respectively. Whereas the 20S core particle is composed of four seven-membered α- and β-rings, the 19S regulatory particle comprises subunits for recognition, unfolding, and translocation of protein substrates. The catalytic activity of the 26S proteasome is ensured by the three standard active site subunits β1, β2, and β5, encoded by the *PSMB6*, *PSMB7*, and *PSMB5* genes, respectively. In immune cells and/or other cell types under stress situations, the standard subunits are replaced by the preferential incorporation of alternative catalytic subunits β5i, β1i, and β2i, encoded by the stress induced genes *PSMB8*, *PSMB9*, and *PSMB10*, respectively (6). Proteasomes containing β1i, β2i, and β5i were initially named immunoproteasomes, because of their potential to modulate the supply of major histocompatibility complex (MHC) class I-derived antigenic peptides. However, immunoproteasomes play critical roles beyond MHC class I antigen presentation that include regulation of gene expression, signal transduction, and/or cell proliferation (7).

The UPS intersects with many critical pathways that regulate cellular processes from energy metabolism to cell proliferation (8), and perturbations of the UPS can dramatically impact on cell fate. Over the past few years, an increasing number of loss-of-function variants in genes coding for UPS components have been identified; disease-causing proteasome variants lead to an expanding clinical spectrum of chronic atypical neutrophilic dermatosis with lipodystrophy and elevated temperature (CANDLE) syndrome (9, 10). As these patients typically show proteasome defects and recurrent episodes of inflammation, these disorders were later referred to as proteasome-associated autoinflammatory syndromes (PRAAS) (11). Monogenic and digenic causes of CANDLE/PRAAS referenced in Online Mendelian Inheritance in Man (OMIM) predominantly include variants in genes encoding subunits of the 20S proteasome core particle. Homozygous or compound heterozygous mutations in *PSMB8*, *PSMB9*, *PSMB4*, or *PSMB10 cause* PRAAS1 (MIM # 256040), PRAAS3 (MIM # 617591), or PRAAS5 (MIM # 619175), respectively, and *PSMA3* mutations are found in digenic inheritance with *PSMB8.* Dominant negative mutations in proteasome assembly factors including *POMP* and heterozygous variants in *PSMG2* cause PRAAS2 (MIM # 618048) and PRAAS4 (MIM # 619183), respectively (11–18). A common pathological feature of CANDLE/PRAAS is the presence of a sterile type I interferon (IFN) gene signature with persistent expression of typical IFN-induced genes (ISGs) in peripheral blood (19, 20). A pathogenic role of type I IFN in CANDLE/PRAAS has been validated by blocking type I IFN signaling with Janus kinase (JAK) 1/2 (JAK1/2)-specific tyrosine kinase inhibitors, which results in clinical remission in 50% of patients associated with normalization of the IFN scores in these patients with mostly *PSMB8* mutations (21, 22). More recently, hematopoietic stem cell transplantation has been reported to be successful in patients with PRAAS harboring loss-of-function mutations in *POMP* or *PSMB4* (23, 24). We here report and functionally characterize eight disease-associated variants in four proteasome genes (*PSMA5*, *PSMC5*, *PSMB10*, and *PSMB8*) in 5 unrelated patients with CANDLE/PRAAS phenotypes; 6 proteasome gene variants have not previously been reported. Two variants were found in the genes *PSMA5* and *PSMC5* with the *PSMA5* variant being most likely disease-causing. Our findings will improve the ability to make an early genetic diagnosis and improved disease management

## Materials and methods

### Research subjects

Written informed consent was obtained from the patient or parents of all subjects who were enrolled into National Institutes of Health (NIH) Institutional Review Board (IRB) approved protocol NCT02974595.

### DNA extraction

DNA from the proband and both parents was extracted from peripheral blood leukocytes using the Gentra Puregene DNA purification kit (Qiagen^®^, Hilden, Germany) according to the manufacturer’s instructions.

### RNA extraction

Patients’ whole blood was drawn into RNA PAXgene tubes, and total RNA was extracted using the PAXgene Blood RNA Kit IVD (Qiagen^®^, Hilden, Germany).

### Determination of type I interferon response gene signature

Gene expression of selected genes was determined by NanoString (NanoString Technologies, Seattle, WA), and a 28-gene type I IFN score was calculated as previously described (25). Briefly, the 28-gene type I IFN score is the sum of the z-scores of 28 type I IFN response genes. Individual gene z-scores were calculated using the mean and standard deviation of the NanoString counts from 19 healthy controls. An IFN signature was considered positive if their value was higher than the 95th percentile of healthy controls (>25.2).

### Whole exome sequencing

For Pts. 2 and 3, exome capture was conducted using the Illumina Exome or Illumina Exome with Enrichment (FLEX) library preparation kits according to the manufacturer’s instructions. Sequencing was performed using an Illumina HiSeq 2500 platform. Alignment to the GRCh37.75/hg19 reference genome and variant calling was performed using Sentieon (sentieon-genomics-201711 or sentieon-genomics-202010) Burrows–Wheeler Aligner (BWA)–maximal exact match (MEM) and Genome Analysis ToolKit (GATK) compliant pipelines on the NIH Biowulf High Performance Cluster. Variants were annotated and prioritized using ANNOVAR (041618 or 060820). For patient 4, exome capture was conducted in a clinical setting using Agilent Clinical Research Exome v1, and sequencing was performed using an Illumina HiSeq platform. Exome data were aligned to the GRCh37.75/hg19 reference genome using the BWA (version 0.7. 17-r1188), and variants were called following the best practices of the Broad Institute using the GATK (version 3. 8-0-ge9d806836) software and annotated using Variant Effect Predictor (version 88.14). All exomes met a minimum of 95% of target bases covered at >10x. Selected variants were validated by Sanger sequencing in the proband and parents.

### Molecular modeling

The mapping of the various α5, β5i/LMP7, β2i/MECL1, and Rpt6 variants within proteasome complexes was realized using the structures of the 20S mouse immunoproteasome (PDB, entry code 3UNH), the human immunoproteasome 20S particle (PDB, entry code 6E5B), and the 19S regulatory particle (PDB, entry code 5LN3) using the freely available Swiss-PdbViewer software.

### Plasmid constructs and transient transfection in HeLa cells

Human PSMB8, PSMA5, and PSMB10 were amplified by PCR from preexisting pIVEX2.3-based constructs and subcloned into the pcDNA3.1/V5-HIS TOPO expression vector (Thermo Fisher Scientific). Human PSMC5 was PCR-amplified from cDNA generated from HeLa cells with HA-tagged primers and cloned into pcDNA3.1/Zeo(+) expression vector (Invitrogen) to generate a N-terminally Hemagglutinin (HA)-tagged PSMC5/Rpt6 protein. Site-directed mutagenesis was used to generate V5/HIS C-terminally tagged variants of β5i/LMP7 (i.e., p.Gln55*, p.Thr75Met, and p.Ser118Pro), β2i/MECL1 (i.e., p.Phe14del and p.Gly167Asp), and α5 (i.e., p.Arg168*). The C-terminus of the (Rpt1-6) ATPase subunits of the 19S regulatory particle mediates the association with the 20S core particle (26–28), and the p.Ala324_Lys360del PSMC5/Rpt6 variant devoid of exon 10 was tagged at the N-terminus with a HA-tandem repeat. Transient transfection of HeLa cells was performed using JetPRIME reagent (Polyplus) following the supplier’s instructions.

### Native-PAGE and Western blotting

At 24-h post-transfection, HeLa cells were lysed in a Tris Salt DTT Glycerol (TSDG) buffer containing 10 mM Tris pH 7.0, 10 mM NaCl, 25 mM KCl, 1.1 mM MgCl_2_, 0.1 mM ethylendiaminetetraacetate (EDTA), 2 mM dithiothreitol (DTT), 2 mM ATP, and 20% glycerol prior to proteins extraction under non-denaturing conditions using five cycles of freeze/thawing in liquid nitrogen. Soluble whole-cell extracts were subjected to protein quantification using a standard Bradford assay and 20 µg of proteins lysates were loaded on 3%–12% gradient bis-tris gels (Thermo Fisher Scientific) at 45 V overnight at 4°C in 50 mM bis-Tris and 50 mM tricine (pH 6,8). Two hundred nanograms of a mixture of purified 20S and 26S proteasomes from erythrocytes (kind gift of Prof. B. Dahlmann, Charité Universitätsmedizin Berlin) were loaded as internal controls. Proteasome native complexes were visualized by in-gel overlay assay by exposing with 0.1 mM of the suc-LLVY-AMC fluorogenic peptide (Bachem) at 37°C for 20 min in an overlay buffer [20 mM tris and 5 mM MgCl_2_ (pH 7.0)]. Proteasome bands were visualized by exposing gels to UV light at 360 nm and detected at 460 nm using an Imager. Native protein complexes were the transferred to Polyvinylidene difluoride (PVDF) membranes (200 V for 1 h), which were subsequently blocked and probed with primary antibodies specific for V5 and HA.

### SDS-PAGE and Western blotting

At 24-h post-transfection, HeLa cells were lysed in in equal amounts of TSDG buffer containing 10 mM tris (pH 7.0), 10 mM NaCl, 25 mM KCl, 1.1 mM MgCl_2_, 0.1 mM EDTA, 2 mM DTT, 2 mM ATP, and 20% glycerol. Protein lysates were quantified using a standard Bradford assay and separated by 10% or 12.5% Sodium dodecylsulfate (SDS)-PAGE prior to transfer to PVDF membranes (200 V for 1 h). Following blocking (20-min exposure to 1× Roti^®^-Block at room temperature), membranes were probed with primary antibodies directed against V5 (Invitrogen, clone 46-O705), β5i/LMP7 (Santa Cruz Biotechnology, clone A7), α5 (Enzo Life Sciences, Clone MCP196), Rpt6 (Enzo Life Sciences, clone p45-110), HA (BioLegend, clone HA.11), and β-actin (Santa Cruz Biotechnology, clone C4) overnight at 4°C under mild shaking. Bound antibodies were visualized using Horseradish peroxidase (HRP)-coupled anti-mouse and anti-rabbit secondary antibodies and Enhanced chemiluminescence (ECL) chemiluminescence (Bio-Rad).

## Results

### Case reports and identification of six new proteasome variants

Patients 1 to 4 have not previously been reported. DNA of all subjects with clinical CANDLE-like features and their parents was screened for known and potentially novel proteasome variants by whole exome sequencing (WES).

**Figure 1:**
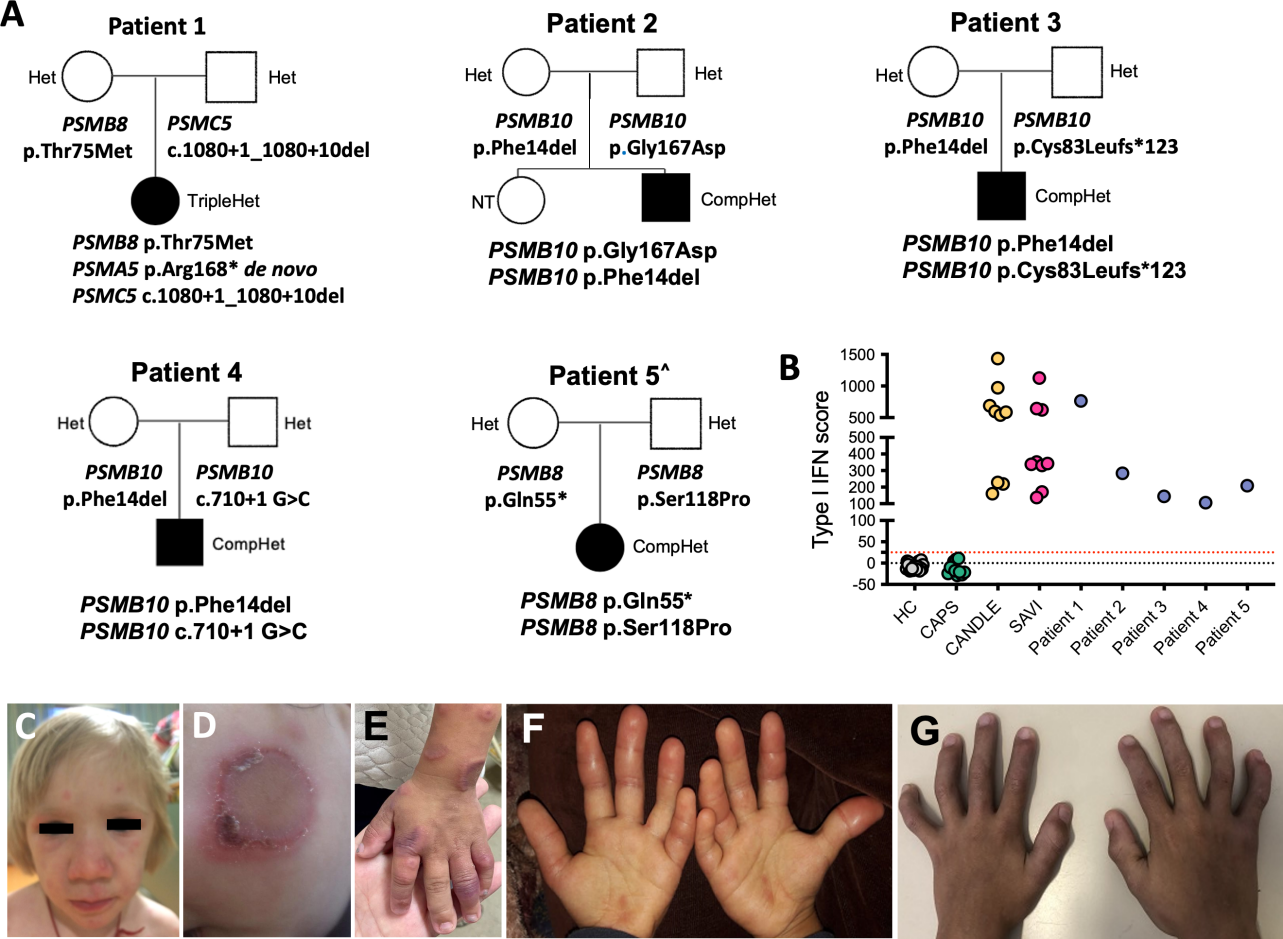
Clinical features and pedigrees. **(A)** Pedigrees of five families (patients 1–5) showing digenic or trigenic inheritance for patient 1 who harbors a novel *de novo* variant in *PSMA5* and a rare *PSMC5* variant on the paternal and a known *PSMB8* mutation on the maternal allele. Three unrelated Brazilian patients present with four novel *PSMB10* variants. All share the p.Phe14del variant plus a different novel variant. Open squares and circles represent healthy males and females, respectively, whereas black squares and circles represent PRAAS affected males and females, respectively. ^ Patient 5 was previously reported (29). **(B)** The IFN score obtained from whole blood prior to treatment with JAKis was assessed by NanoString and presented as the sum of z-scores of 28 ISGs. Type I IFN scores for the five patients with PRAAS are depicted in comparison with healthy controls (HC), patients with the IL-1–mediated disease cryopyrin-associated periodic syndrome (CAPS), and patients with the type I interferonopathies chronic atypical neutrophilic dermatosis with lipodystrophy and elevated temperature (CANDLE) and stimulator of interferon genes (STING)–associated vasculopathy with onset in infancy (SAVI). Type I IFN score (mean ± SD): HC, −4.8 ± 9.53; NOMID, −9.37 ± 10.97; CANDLE, 738.2 ± 754.4; SAVI, 495.6 ± 225.9; patient 1, 763.27; patient 2, 283.57; patient 3, 144.76; patient 4, 106.75; patient 5, 209.47. Clinical skin and joint features in patients with CANDLE/PRAAS are shown in **(C–G)**. **(C)** Facial features of nodular rash over cheeks and forehead and periorbital edema and swelling with mild lipoatrophy in temple and cheek area (Pt. 1). **(D)** Annular erythematous skin rash on left cheek at 9 months of age (Pt. 3). **(E)** Annular erythematous lesions involving the hand and arm at 12 months of age (Pt. 3). **(F)** Erythematous swollen fingers at the age of 3 years (Pt. 4). **(G)** Fixed flexion contractures of the interphalangeal joints at the age of 5 years (Pt. 4).

Patient 1 [variants in *PSMB8*, *PSMA5* (*de novo*), and *PSMC5*] is a 6-year-old Caucasian girl born to healthy, non-consanguineous parents presented with redness swelling of the right heel that later extended to the second toe of the right foot at 2 weeks of life. At 2 months of age, she developed fevers and generalized skin eruptions on her nose, feet, and wrists. Laboratory exams showed anemia with hemoglobin of 9.0 g/dl, leukocytosis with a white blood cell count of 20,000 cells/ml, and elevated erythrocyte sedimentation rate (ESR) at 58 mm/h. The patient was started on prednisolone, and the symptoms improved. At 6 months of age, the skin eruption recurred with nodular rash spreading to the face, trunk, and extremities. Hydroxychloroquine was added to prednisolone which improved rashes. At 7 months, the development of hepatosplenomegaly and continued generalized rashes prompted a skin biopsy that was suggestive of erythema nodosum, which, together with elevated acute phase reactants and lactate dehydrogenase (LDH), led to a clinical diagnosis of possibly CANDLE. Genetic testing revealed a known *PSMB8* founder heterozygous mutation, p.Thr 75Met, that she inherited from her mother, which does not explain her disease (**Figure 1A**). Treatment with azathioprine, interleukin 6 (IL-6) inhibitor tocilizumab and methotrexate resulted in only temporary improvement. After 1 month of tocilizumab treatment, she refused to stand up on her feet due to panniculitis of her heels. Recurrent panniculitis around eyelids, shin, and forearms, in association with leukocytosis, and elevated ESR and C-reactive protein (CRP) led to treatment with treatment with JAK inhibitor (JAKi), tofacitinib at 4 years of age. Tofacitinib was increased to 20 mg/day. At 5 years of age, she experienced another episode of flare with generalized skin eruption and increased CRP, which was treated by increasing the tofacitinib dose to 25 mg/day, and tocilizumab was added back. She is currently on combination therapy of tofacitinib 30 mg/day (0.7 mg/kg) and tocilizumab 162 mg (3.8 mg/kg per dose) every 2 weeks. Her disease is in remission with rare occurrence of breakthrough episodic rash (**Figure 1C**).

Patient 1 harbored a trigenic pattern of heterozygous mutations including in *PSMB8* and *PSMA5* encoding the 20S core subunits β5i/LMP7, and α5, respectively, and in *PSMC5* encoding the Rpt6 subunit of the 19S regulatory particle, (**Figure 1A**). She harbors the most common Spanish/Portuguese/Hispanic founder mutation in *PSMB8*, p.Thr75Met (11), inherited from her mother (**Figure 1A**). A truncating variant in *PSMA5* (p.Arg168*) that results in an α5 that lacks 73 C-terminal amino acids occurred *de novo*, and a splice-site variant in *PSMC5* (c.1080 + 1_1080 + 10del) that results in an exon 10–skipped Rpt6 was inherited from her father (**Supplementary Figures 1C, D**).

Patients 2, 3, and 4 are compound heterozygous for variants in *PSMB10* encoding the immunoproteasome subunit β2i/Multicatalytic Endopeptidase Complex (MECL1). A different mutation at amino acid position p.Phe14, p.Phe14Ser (16), has previously been reported in homozygosity in an Algerian boy; the p. Phe14del mutation that is present in all three Brazilian patients is novel and has not been reported in public databases but affects the same amino acid position (**Figure 1A**).

Patient 2 (two novel *PSMB10* variants), a 3.5-year-old Brazilian boy, presented in the perinatal period with heterogeneous annular-shaped ulcerated papulopustular skin lesions on his face, hands, and feet (**Figures 1D, E**). A skin biopsy revealed neutrophilic dermatosis with dermal mucinosis. Initiation of prednisolone 1 mg/kg/day resulted in initial clinical improvement, but lesions relapsed with weaning of steroids. At the age of 6 months, he developed daily fevers and at the age of 8 months, erythematous periorbital edema, failure to thrive (below the third percentile of weight and height), and neurodevelopmental delay (NDD) were noted. He had microcytic hypochromic anemia, leukocytosis with neutrophilia, thrombocytosis, elevated acute phase reactants (erythrocyte ESR and CRP), as well elevated serum levels of ferritin, liver, and muscle enzymes and hypertriglyceridemia. Infectious etiologies and hematological malignancies were ruled out. Colchicine (0.03 mg/kg/day) and prednisolone (0.5 mg/kg/day) were initiated at 8 months of age, but, despite the initial improvement of skin lesions and reduction of fever spikes, the patient relapsed after 12 months of treatment. At the age of 20 months, he was clinically suspected to have CANDLE and when genetically confirmed, the JAKi tofacitinib was initiated at 0.9 mg/kg/day with partial improvement or fever and skin lesions as well as decrease of acute phase reactants. Currently, at the age of 3.5 years, the patient still presents with recurrent episodes of skin rash and systemic inflammation (CRP of 33 mg/L) and prednisolone dose is at 0.44 mg/kg/day. Switching tofacitinib to baricitinib is thus being considered. The p.Gly167Asp variant in patient 2, which he inherited from his asymptomatic father, has been reported in one heterozygous subject in the gnomAD database (7) (**Figure 1A** and **Supplementary Figure 2A**).

Patient 3 (additional novel *PSMB10* variant) is a 6.5-year-old Brazilian boy who presented with recurrent episodes of annular erythematous diffuse skin lesions predominantly on his face and extremities since 15 days of life. A skin biopsy revealed a non-specific perivascular lymphocytic infiltrate with dermal mucinosis. He had poor weight gain and developed facial lipoatrophy, frontal bossing, and erythematous and edematous fingers. At the age of 3 years, he developed recurrent fevers. At the age of 5 years, lipodystrophy and fixed flexion contractures of interphalangeal joints were noticed (**Figures 1F, G**). He does not have neurodevelopment delay, muscle pain, or weakness. Laboratory evaluation showed microcytic anemia, elevated acute-phase reactants (ESR and CRP), hypertriglyceridemia, and elevated hepatic and muscle enzymes. A muscle biopsy revealed irregular muscle fibers (atrophic and hypertrophic), without inflammatory infiltrate. Infectious etiologies and hematological malignancies were ruled out. The patient had a partial response to prednisolone (2 mg/kg/day) and methotrexate (10 mg/m^2^) with improvement of arthritis and decrease of acute phase reactants and muscle enzymes, but, due to persistency of recurrent episodes of fever and panniculitis, baricitinib (2 mg/day, 0.1 mg/kg/day) was started at the age of 6 years. Prednisolone was progressively weaned and stopped, and, currently, at the age of 6 years and 10 months, the patient is on clinical remission on baricitinib and methotrexate therapy. Patient 3 harbors another novel *PSMB10* variant, a frameshift mutation p.Cys83Leufs*123 that he inherited from his asymptomatic father. The variant has been reported in gnomAD in 28 heterozygous subjects of predominantly African American ancestry (**Figure 1A** and **Supplementary Figure 2A**).

Patient 4 (additional novel *PSMB10* variant), is a 2-year-old Brazilian boy of non-consanguineous parents who presented with persistent violaceous skin lesions and recurrent fever since the age of 10 days of life. Laboratory workup showed elevated acute-phase reactants (CRP and ESR), elevated liver enzymes (200–400 U/L), and imaging demonstrated sparse liver calcifications, and cerebellar vermis hypoplasia. Skin biopsies showed neutrophilic dermatosis. Treatment with oral prednisolone (1 mg/kg/day) was started; however, fever and skin lesions recurred when tapering prednisolone. The patient developed complications of chronic systemic corticosteroid use, including hirsutism, truncal obesity, and poor growth. Colchicine and dapsone had no effect on symptoms. Genetic screening using a primary immunodeficiency targeted gene panel was inconclusive, and WES was performed at the age of 1 year and 5 months, which revealed two novel *PSMB10* variants (c.40_42del and p.Phe14del, which are also present in patients 2 and 3) and a third novel *PSMB10* splice site variant (c.710 + 1G>C) (**Figure 1A**). Baricitinib 3 mg/day was started (0.3 mg/kg/day) at 1 year and 8 months old with complete suppression of skin lesions and fever, as well as normalization of liver enzymes and CRP/ESR. Corticosteroid was slowly tapered and discontinued after 6 months on baricitinib. Currently, at 2 years and 1 month old, the patient is in clinical remission, and no treatment side effects were observed so far.

Patient 5 has been previously reported (29); a concise description of her disease can be found in the supplements of this paper. The patient harbors two previously reported heterozygous *PSMB8* variants (p.Ser118Pro and p.Gln55*) (29) (**Figure 1A**).

An elevated type I IFN score was found in all five patients, and the scores were comparable to that of other patients with CANDLE/PRAAS or STING associated vasculopathy with onset in infancy (**Figure 1B**).

### Structural mapping of the six new proteasome variants

Most of the novel proteasome alterations identified in the subjects were highly evolutionary conserved across species from human to zebrafish (**Supplementary Figure 3D**).

**Figure 2:**
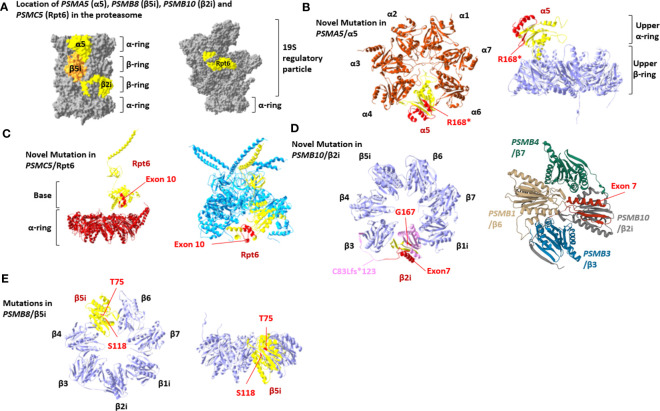
Proteasome variant positions within the 26S proteasome complex. **(A)** A lateral view of the 20S core particle (left) and 19S regulatory particle (right), indicating the localization of the *PSMA5*/α5, *PSMB10*/β2i, *PSMC5*/Rpt6 (yellow), and *PSMB8*/β5i (orange) affected subunits. **(B)** Left: bottom view of the upper 20S proteasome α-ring (brown) showing the localization of the missing C-terminal part of the p.(Arg168*) *PSMA5*/α5 variant (yellow). Right: Lateral view of the upper 20S proteasome β-ring (purple) together with the p.(Arg168*) *PSMA5*/α5 variant (yellow) from the upper α-ring. **(C)** Left: Lateral view of the 20S proteasome α-ring (brown) together with PSMC5/Rpt6 (yellow) and its exon 10 (red) from the 19S regulatory particle. Right: Lateral view of the of the ring base of 19S regulatory particle (blue) containing the PSMC5/Rpt6 with exon 10 marked in red. **(D)** Left: Top view of the lower 20S proteasome β-ring (purple) showing the localization of the G167 residue, exon 7 (red) within the PSMB10/β2i subunit (yellow). The theoretical frameshift PSMB10/β2i variant emerging from the c.247_248insT alteration is highlighted in pink. Right: View of the 20S immunoproteasome subunit β2i (dark gray) together with the β6, β3, and β7 of the proteasome β-ring showing the localization of β2i exon7 (red). **(E)** Top (left) and lateral (right) views of the lower 20S proteasome β-ring showing the localization of the T75 and S118 residues (marked in red) within the β5i (PSMB8) subunits (yellow).

Three-dimensional, spatial *in silico* mapping of all mutated residues within the 20S and 19S particles (30) (PDB, entry code 3UNH) (31) (PDB, entry code 5LN3) shows the physical interaction sites of β5i/LMP7/*PSMB8* and α5/*PSMA5* at the α/β-ring interface (**Figure 2A**). The most frequent *PSMB8* mutation, p.Thr75Met, maps to the first β-sheet of β5i/LMP7, outside of the inter-subunit joining area (**Figure 2E**). Similarly, the 73 C-terminal amino acids in the α5 subunit, which are deleted with the *PSMA5* p.Arg168* mutation, do not affect binding of neighboring α- and β-subunits (**Figure 2B**). However, the *PSMA5*/α5 lacks the C-terminal α-helices 4 and 5, which are predicted to bind *PSMC2*/Rpt1 ATPase of the 19S regulatory particle (**Supplementary Figure 3B**). Pt.1 also harbors an intronic 10-nucleotide deletion at a canonical splice site in *PSMC5*, that results in exon 10 skipping in *PSMC5/*Rpt6 Although the level of exon 10 skipping in the patient is not known the structural implications have been modeled. As shown in **Figure 2C**, the C-terminal three α-helices encoded by this region are not in close proximity to any ATPases or α-subunits, suggesting that the potential incorporation of this variant into 26S proteasomes might not significantly affect the structure of the 19S regulator particle or its association with 20S core particles.

All Brazilian patients (Pt. 2–4) carry the *PSMB10*/β2i/MECL1 p.Phe14del variant that localizes to the pro-peptide, which is typically cleaved off during processing and not incorporated into mature proteasomes (**Supplementary Figure 3C**). A second *PSMB10*/β2i/MECL1 variant, p.Gly167Asp (Pt. 2), localizes to the ninth β-sheet of the processed subunit and does not generate direct polar bonds with the neighboring β1i and β3 subunits of the same β-ring or the β4 subunit from the subjacent β-ring.

The mutant RNA transcript for *PSMB10* p.Cys83Leufs*123 mutation (Pt. 3) undergoes nonsense mediated decay, and no protein is produced (**Supplementary Figures 2B, C**). The mutation is, therefore, not further evaluated.

The c.710 + 1 G>C splice site mutation (Pt. 4) is predicted to splice out exon 7 of *PSMB10*. This exon encodes a 51–amino acid sequence composed of one α-helix, two antiparallel β-sheets, and a C-terminal loop that are forming the external surface of the β-ring without contact to the neighboring β-subunits of the same β-ring except for β3 (**Figure 2D**, left panel). These structures also contain interface residues jutting inside the adjacent β-ring, particularly those of the α-helix and the C-terminal loop that are directly exposed to the β7 and β6 subunits of the other β-ring, respectively (**Figure 2D**, right panel). Structural modeling suggests that loss of exon 7 might severely affect the dimerization of the 16S precursor complexes that prevent incorporation of PSMB10/β2i/MECL1 into the mature proteasome.

Of the two disease-causing *PSMB8*/β5i/LMP7 variants in patient 5, protein subunits containing the p.Ser118 Pro variant that localizes to the sixth β-sheet are not in direct contact with neighboring β4-, β9-, or the α- subunits (**Figure 2E**). The proline substitution likely destroys the secondary structures by disrupting the sixth β-sheet (**Figure 2E**).

### Functional characterization of the identified proteasome variants

**Figure 3:**
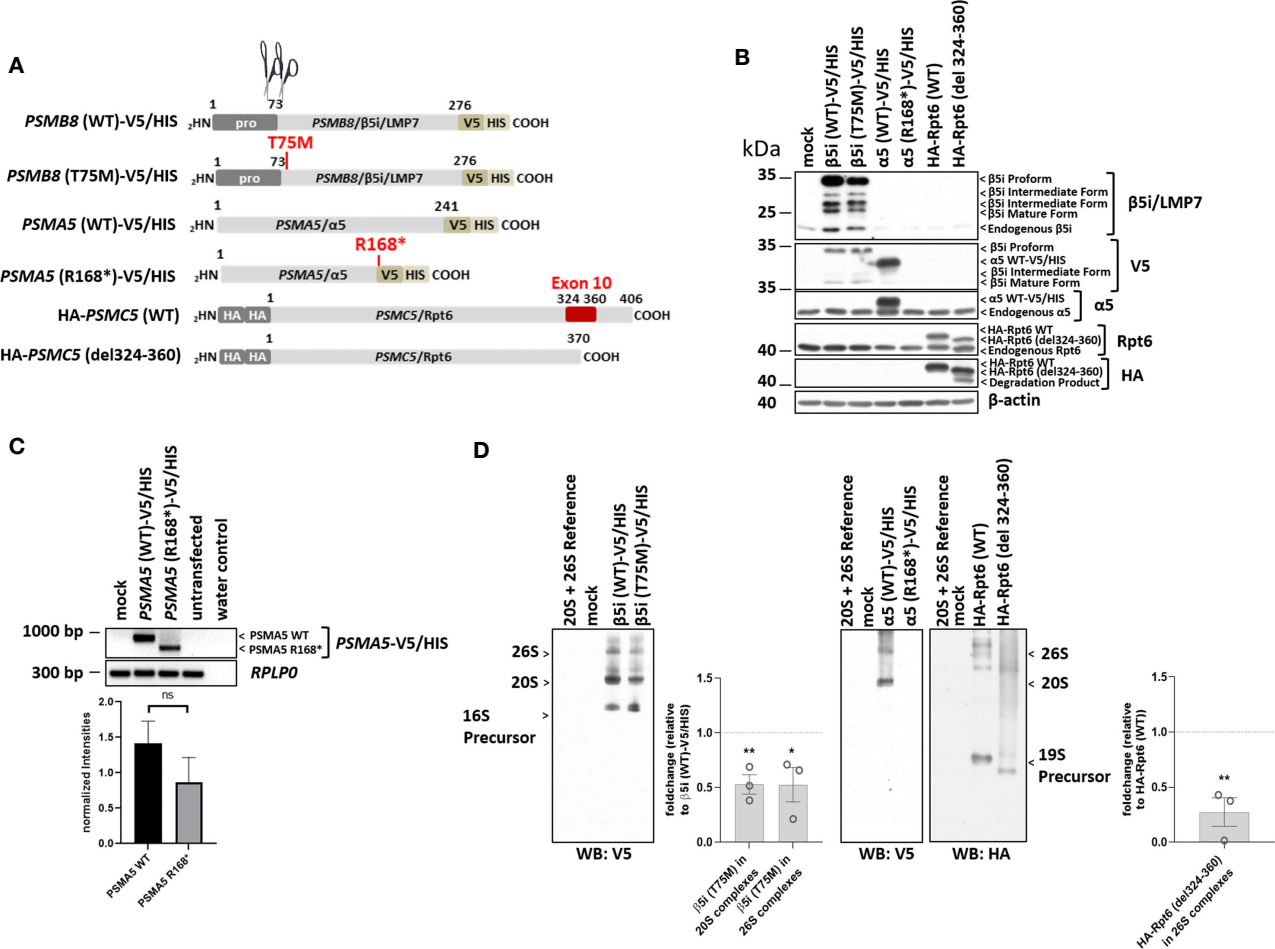
The proteasome subunit variants PSMB8/β5i, p.Thr75Met, PSMA5/α5, p.Arg168*, and PSMC5/Rpt6 p.Ala324_Lys360del (patient 1) are not fully incorporated into 20S/26S proteasome complexes. **(A)** Schematic representation of the *PSMB8*, *PSMA5*, and *PSMC5* constructs encoding wild-type (WT) and β5i/LMP7, α5, or Rpt6 variants, respectively, used in this study. Unlike the *PSMB8* and *PSMA5* constructs that are C-terminally tagged with a combined V5/HIS epitope, the *PSMC5* constructs are N-terminally fused with a HA-tandem repeat. Scissors represent propeptide processing, which does not occur for α5 or Rpt6 variants. **(B)** HeLa cells expressing WT or β5i/LMP7, α5, or Rpt6 variants were subjected to denaturing protein extraction prior to SDS-PAGE/Western blotting analysis using antibodies specific for β5i/LMP7, α5, Rpt6, V5, *myc*, and β-actin (loading control), as indicated. **(C)** HeLa cells transiently transfected with WT or variants of the α5 proteasome subunit were assessed for their content in α5-V5/HIS transcripts by RT-PCR using specific primers. Equal sample loading was ensured by amplifying the *RPLP0* housekeeping gene, as indicated. The quantification of the *PSMA5* bands from three replicates was normalized using the obtained *RPLP0* housekeeping gene signals and subsequently analyzed and visualized using GraphPad software (GraphPad Software, Inc., California, USA). **(D)** HeLa cells engineered to express WT and variants of the β5i/LMP7, α5, or Rpt6 subunits were subjected to non-denaturing protein extraction and subsequently analyzed for their amounts of V5- and HA-containing proteasome complexes by native-PAGE/Western blotting, as indicated. Densitometric evaluation (n = 3) of impaired incorporation of β5i/LMP7 T75M-V5 (p = 0.006 for incorporation into 20S complexes; p = 0.04 for incorporation into 26S complexes) or HA-Rpt6del324-360 variants (p = 0.005) in relation to the WT as fold change. WT incorporation was set to 1. ns, not significant; * p-value <0.05; ** p < 0.01.

**Figure 4:**
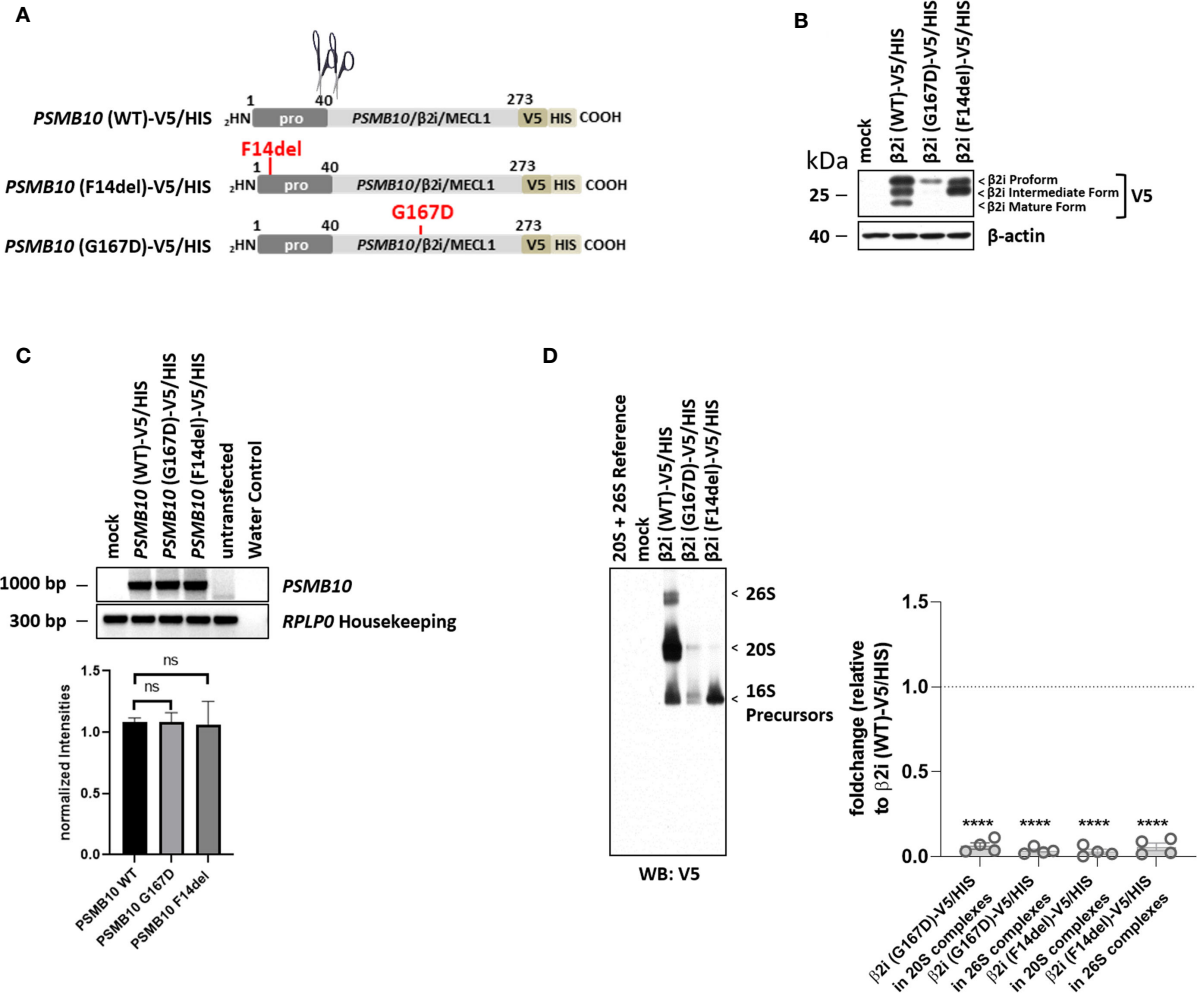
Both PSMB10/β2i variants, p.Phe14del and p.Gly167Asp (patient 2), cannot mature and incorporated. **(A)** Cartoon depicting the C-terminally V5/HIS-tagged *PSMB10* constructs encoding wild-type (WT) and β2i/MECL1 variants, as indicated. Scissors represent propeptide processing. **(B)** HeLa cells engineered to express WT and β2i/MECL1 variants were subjected to protein extraction prior to Western-blot analysis using antibodies specific for V5 and β-actin (loading control). **(C)** HeLa cells ectopically expressing WT and *PSMB10* variants were subjected to RNA extraction and subsequent RT-PCR using primers specific for the overexpressed *PSMB10* species, as indicated. Amplification of *RPLP0* housekeeping was used a control to ensure equal loading of samples. The quantification of the *PSMB10* bands from three replicates was normalized using the obtained *RPLP0* housekeeping gene signals and subsequently analyzed and visualized using GraphPad software (GraphPad Software, Inc., California, USA). **(D)** HeLa cells transfected with WT and β2i/MECL1 variants were assessed for their content of V5-containing native complexes by native-PAGE/Western blotting using an antibody directed against V5, as indicated. Densitometric evaluation (n = 3) of failed incorporation of β2i/Mecl-1 variants into 20S or 26S complexes in relation to the WT variants as fold change (p-values < 0.0001). WT incorporation was set to 1. ns, not significant; **** p-value <0.0001.

**Figure 5:**
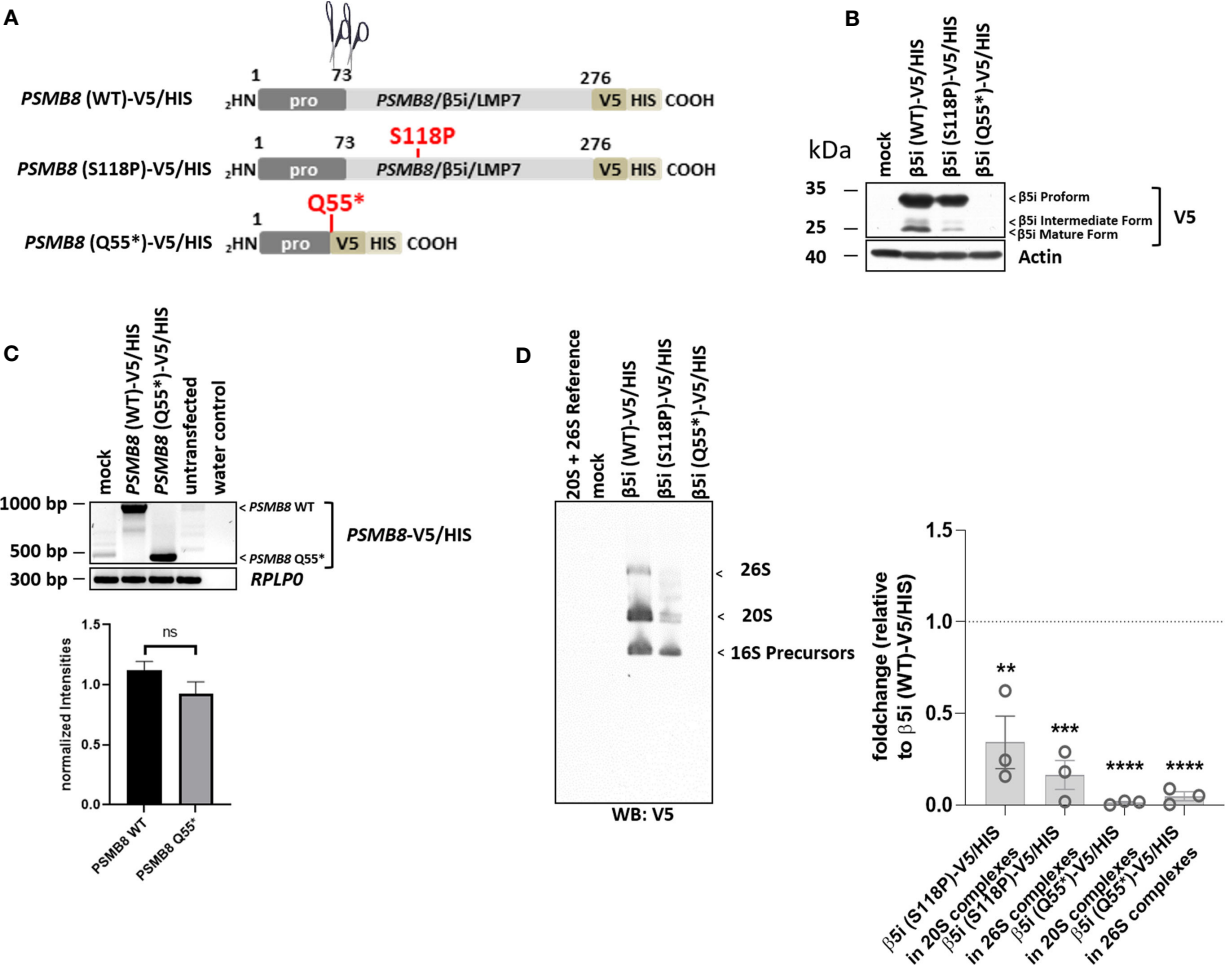
Both *PSMB8* variants, p.Gln55* and p.Ser118Pro (patient 5), fail to assemble into mature 20S and 26S proteasomes. **(A)**. Depiction of the *PSMB8* wild-type and mutant constructs shows the amino acids (AA) with in the prodomain and the functional domain of PSMB8/β5i/LMP7. All constructs were fused at the C-terminus in frame with a combination of V5 and HIS tags, as indicated. Scissors represent propeptide processing. **(B)** HeLa cells transiently transfected with *PSMB8* constructs were assessed for their V5 content by Western blotting, as indicated. Equal protein loading was ensured by probing the membrane with a monoclonal antibody specific for β-actin. **(C)** HeLa cells engineered to express wild-type and PSMB8 variants were subjected to RNA extraction and subsequent RT-PCR using primers specific for the overexpressed PSMB8 species, as indicated. Amplification of *RPLP0* housekeeping was used a control to ensure equal loading of samples. The quantification of the *PSMB8* bands from three replicates was normalized using the obtained *RPLP0* housekeeping gene signals and subsequently analyzed and visualized using GraphPad software (GraphPad Software, Inc., California, USA). **(D)** HeLa cells expressing wild-type or mutant *PSMB8* were subjected to non-denaturing protein extraction prior to native-PAGE analysis by Western blotting using an antibody specific for V5, as indicated. Densitometric evaluation (n = 3) of failed incorporation of β5i/LMP7 S118P-V5 or β5i/LMP7 Q55*-V5 variants into 20S complexes or 26S complexes in relation to the WT as fold change (β5i S118P-V5: 20S incorporation, p = 0.01; 26S incorporation, p = 0.0004; β5i Q55*-V5: p-values < 0.0001 for 20S and 26S incorporation). WT incorporation was set to 1. ns, not significant; ** p < 0.01; *** p < 0.001; **** p < 0.0001.

For functional characterization of the identified proteasome variants, there was no material from patients available. To assess the effect of the identified genomic variants on proteasome formation, we therefore ectopically expressed epitope-tagged proteasome subunits as wild-type (WT) or mutant versions in HeLa cells (see schematic representation in **Figures 3**-**5A**) and analyzed protein expression and incorporation into proteasome complexes (**Figures 3**-**5B, D**).

As previously shown, the *PSMB8*/β5i/LMP7 variant, p.Thr75Met (Pt. 1) was expressed but matured less efficiently (**Figure 3B**) and incorporation into 20S/26S complexes was decreased (**Figure 3D**) (11). The *PSMA5*/α5 truncation, p.Arg168* (Pt. 1), yielded messenger RNA (mRNA) levels lower than the WT transcript (not statistically significant) (**Figure 3C**); however, mutant PSMA5/α5 protein was not expressed and incorporated (**Figures 3B, D**), suggesting nonsense-mediated decay of the mRNA.

The *PSMC5*/Rpt6 variant was assessed by generating a construct that lacked exon 10, p.Ala324_Lys360del (Pt. 1). Sequencing of cDNA from Pt. 1 confirmed skipping of exon 10 in *PSMC5* and mRNA lacking exon 10 (**Supplementary Figures 1C, D**). The resulting PSMC5/Rpt6 protein variant, p.Ala324_Lys360del, was expressed at levels similar to WT but the mutant protein was not efficiently incorporated into the mature 26S proteasome complexes. Reduced incorporation iresults in formation of misassembled precursor complexes of the 19S regulatory particle and in an assembly defect of the 26S proteasome complexes.

The *PSMB10*/β2i/MECL1 p.Phe14del variant (Pts. 2 to 4) was efficiently expressed as a precursor subunit but was not incorporated into mature proteasomes, whereas the p.Gly167Asp variant was less efficiently expressed at the protein level in HeLa cells (**Figure 4B**). Neither of the two variants resulted in changes in transcription efficiency (**Figure 4C**). Compared to WT, both mutant protein subunits were incorported into precursor complexes but failed to get efficiently incorporated into the 20S and 26S complexes thus pointing to maturation impairment.

The previously reported *PSMB8*/β5i/LMP7 variant, p.Gln55*, was transcribed at low levels (**Figure 5C**) but failed to generate a functional protein and was, therefore, not incorporated into the proteasome (**Figures 5B, D**). The p.Ser118Pro substitution severely impaired *PSMB8*/β5i/LMP7 processing (**Figure 5B**) that resulted in inefficient incorporation into the mature proteasomes as illustrated by decreased V5 staining in the regions of the 20S and 26S complexes on native-polyacrylamide gel electrophoresis (PAGE) analysis (**Figure 3D**).

## Discussion

In this paper, we report and characterize eight PRAAS-associated proteasome variants, all impact proteasome function. We report a disease-causing mutation in *PSMA5* that occurred de novo and has not previously been reported. We also report four novel disease-causing *PSMB10* variants in three Brazilian patients and functionally characterize two previously reported *PSMB8* variants. A novel paternally inherited splice-site variant in *PSMC5* was detected in the patient who also had a pathogenic maternally inherited variant in *PSMB8* and the de novo *PSMA5* mutation. Without knowing the effect of the *PSMC5* variant on alternative splicing in hematopoietic and non-hematopoietic cells, the clinical impact of this variant cannot be determined.

Following the previously reported pattern of CANDLE/PRAAS-causing mutations, seven of the eight reported variants affect α- and β-subunits of the 20S core particle of the proteasome that, when mutated, cause a CANDLE/PRAAS phenotype. CANDLE/PRAAS presents early in life with rashes and systemic inflammation (**Figures 1C–G**) but with variable disease severity and a genotype-phenotype association with clinical responses to JAK inhibition that are more complete in patients with immunoproteasome mutations in *PSMB8*. The clinical phenotype of patients 2–5 is similar to that of patients with CANDLE/PRAAS with *PSMB8* mutations, with three patients harboring compound heterozygous variants in the immunoproteasome, *PSMB10*, and one in *PSMB8*. Patient 1 who has a mutation in the constitutive proteasome component, *PSMA5*, requires combination treatment of a JAKi with tocilizumab due to more JAKi refractory disease reminiscent of what is seen in patients with mutations in other constitutive proteasome components, *PSMB4* and *PSMA3*; interestingly, the combination has resulted in clinical remission.

The clinically milder phenotypes in patients with mutations in the immunoproteasome components *PSMB8* or *PSMB10* have structural reasons. The immunoproteasome subunits—*PSMB8/*β5i, *PSMB9/*β1i, and *PSMB10/*β2i—can replace their respective constitutive counterparts—*PSMB5*/β5, *PSMB6*/β1, or *PSMB7*/β2, respectively—in the context of inflammation and/or during cell stress, when the demand for increased protein degradation increases and the incorporation of the inducible component increases protein degradation capacity. In the absence of the mutant *PSMB8-* or *PSMB10-*encoded protein subunits, proteasomes are assembled through incorporation of the respective constitutive components. This phenomenon is well established in mouse models with ablation of *PSMB8* (11, 32, 33). In agreement with these findings, variants that result in non-expression or non-incorporation of an immunoproteasome component can occur in homozygosity but would be expected to be embryologically lethal if present in a gene encoding a constitutive proteasome component that cannot be substituted for. In situations when loss-of-function mutations allow for incorporation of a mutant allele with residual function in compound heterozygosity or when present in a digenic inheritance model, the WT protein encoded from the WT allele gets incorporated and forms an altered proteasome that often has reduced but sufficient function to be compatible with life (11). These considerations would further suggest that the novel disease-causing nonsense *PSMA5* variant *PSMA5*/α5 p.Arg168* that does not generate a protein cannot exist in homozygosity. PSMA5/α5 is a constitutive component of the 20S core proteasome, the assembly of which critically depends on the estimated 50% of WT proteins that are encoded from the WT *PSMA5* allele. Similar to other digenic patients, patient 1 harbors a second mutation in *PSMB8*, p.Thr75Met, the most common founder mutation in patients of Hispanic and Spanish and Portuguese ethnicity (**Figure 1A**).

A significant number of CANDLE/PRAAS-causing mutations affect RNA stability that either results in no or reduced protein production. This is seen in the nonsense mutations, *PSMB8*/β5i/LMP7 p.Gln55*and *PSMA5*/α5 p.Arg168*, and the missense mutation in *PSMB10*/β2i, p.Gly167Asp, that all fail to produce sufficient amounts of functional proteins consistent with decreased stability of the mutant RNAs. An allele carrying a frameshift mutation in *PSMB10*/β2i p.Cys83Leufs*123 is not expressed (**Supplementary Table 1**). Another group of mutations impairs cleavage of the pro-peptide and thereby prevents incorporation of the mutant proteasome component. This is seen with the novel *PSMB10* variant, p.Gly167Asp, and the variant p.Phe14del that was present in all three non-related Brazilian patients and their mothers and is likely a founder mutation in Brazilian patients with indigenous ethnicity. Both of these variants are not processed and not incorporated into the 20S and/or 26S proteasome complexes (**Figure 4**). In line with the study by Sarrabay et al. that reported a *PSMB10* p.Phe14Ser variant with impaired pro-peptide cleavage, our data confirm a critical role for amino acid p.Phe14 in pro-peptide processing (16). The mechanisms by which the p.Gly167Asp β2i/MECL1 variant prevents β2i/MECL1 processing are less clear, but the Gly167Asp substitution results in the acquisition of a negative charge that conceivably may disrupt the ninth β-sheet of the subunit.

The *PSMB10* c.710 + 1G>C intronic variant encodes a protein lacking exon 7, which is predicted to interrupt the proteasome assembly by perturbing interactions with three neighboring β-subunits (**Figure 2D**). Our structural modeling of the novel disease-causing variants on proteasome assembly predicted to severely compromise protein homeostasis under challenged conditions when supply of additional proteasome components is limited by the mutant alleles (34).

Interestingly, patient 1 who harbors a *de novo* heterozygous *PSMA5* and a known disease-causing *PSMB8* variant has a third heterozygous splice-site variant in *PSMC5* encoding the Rpt6 ATPase component of the 19S regulatory particle. The *PSMC5* variant, c.1080 + 1_1080 + 10del, yielded a Rpt6 p.Ala324_Lys360del protein variant lacking amino acid positions 324–360 encoded by exon 10 (**Figure 3B**). Although our assays revealed significantly less incorporation of the PSMC5/Rpt6 splice variant into 26S proteasomes (**Figure 3D**), the precise contribution of the PSMC5/Rpt6 p.Ala324_Lys360del splice-site variant to the PRAAS phenotype of Pt. 1 remains unclear. The level of splicing resulting in exon 10 skipping vs wildtype transcription of the mutant PSMC5 allele is currently unknown and may be tissue specific. Furthermore, the wildtype allele may be incorporated at a higher frequency and partially compensate for the defect in vivo. Together with the data from the structural modeling that predicted no major structural impact of exon 10 deletion, this may suggest that this variant is more likely a modifier and its contribution to driving a CANDLE/PRAAS phenotype needs to be evaluated in future patients. 

With the exception of the *PSMC5* variant, our findings agree with the notion that the CANDLE/PRAAS phenotype that presents with prominent neutrophilic panniculitis, systemic inflammation, and, in some instances, cognitive delay is mostly caused by loss-of-function mutations in the 20S proteasome subunits or in proteins that mediate proteasome assembly (11–18, 35). However, heterozygous mostly *de novo* mutations in *PSMD12* coding for the 19S regulatory subunit Rpn5 of the proteasome cause syndromes that mainly present with NDD (36, 37). These observations support a proteasome complex–phenotype association with deficiencies of 19S or 20S complexes causing NDD or PRAAS, respectively (38). This hypothesis was later challenged, as NDD and only few systemic inflammatory findings have been reported in patients carrying mutations in the 20S subunit β6 encoded by *PSMB1* (39). Vice versa, disease-causing variants in *PSMD12* that leads to more prominent NDD were shown to have a blood IFN signature with subclinical inflammation (40, 41). In a recent study, protein kinase R (PKR) was defined as an innate immune sensor for proteotoxic stress via interaction with accumulated IL-24 to promote dysregulated type I IFN signaling (42). Furthermore, we have shown that pathogenic variants of the 19S particle subunit Rpt5 (encoded by *PSMC3*) cause NDD. The proteostatic perturbations in T cells from patients with PSMC3/Rpt5 variants correlated with a dysregulation in type I IFN signaling, which could be blocked by inhibition of PKR. These results indicate that, despite different organ manifestations, the molecular mechanisms driving the clinically different disease manifestations are similar but may act in a tissue specific manner (43). Mechanisms that control severe systemic inflammation versus more prominent neurodevelopmental issues are not well understood, but our findings add additional mutations that can in the future be assessed in organ-specific disease models (i.e., brain models), which may shed light on the tissue-specific organ damage in the spectrum of patients with rare proteasomopathies. Whether proteasomopathy patients with NDD would benefit from JAKis and/or anti–IL-6 treatment remains to be assessed in future studies.

In summary, we identified a disease-causing variant in *PSMA5*, a novel variant in *PSMC5*, which impairs proteasome assembly and four novel *PSMB10* variants in three Brazilian patients that shed light on disease-causing mutations in the PRAAS spectrum of diseases that will improve the diagnosis of CANDLE\PRAAS. We also provide a disease model that reconciles the currently known mutations with disease phenotype.

## Data availability statement

The whole exome sequencing data presented in this study can be found in online repository dbGaP. The link to the repository and accession number can be found below: https://www.ncbi.nlm.nih.gov/gap/, phs001946.v2.p1.

## Ethics statement

The studies involving human participants were reviewed and approved by NIH Institutional Review Board approved protocol NCT02974595 (NIH), 9000 Rockville Pike, Bethesda, Maryland 20892. Written informed consent to participate in this study was provided by the participants’ legal guardian/next of kin. Written informed consent was obtained from the minor(s)’ legal guardian/next of kin for the publication of any potentially identifiable images or data included in this article.

## Author contributions

JP: performed experiments, data analysis, data interpretation, data presentation, molecular modelling, figure design and manuscript revision. SA, DP, AK, MT, AS, MR, KU, AP, FS, ST, MG, AF, NM, MF, RP: Patient care, consultation, and clinical data collection. AR, SP, BL, JS: Performed experiments. JN, SR, SFP: Performed data analysis. AO, CM: Processed sequencing data and provided splice-site predictions. FE: Study design, data interpretation and manuscript writing. RG-M: study design, data interpretation, manuscript revision. AJ: study design, data interpretation, manuscript writing. EK: study design, data interpretation, manuscript revision, funding acquisition. All authors contributed to the article and approved the submitted version.
