# Supplementary material for: Identification of eight novel proteasome variants in five unrelated cases of proteasome-associated autoinflammatory syndromes (PRAAS)
Source: Front Immunol. 2023 Aug 4;14:1190104. doi: 10.3389/fimmu.2023.1190104 (PMC10436547; doi:10.3389/fimmu.2023.1190104)
Supplement: Supplementary file 1 [file DataSheet_1.docx]

**Identification of eight novel proteasome variants in five unrelated cases of proteasome-associated autoinflammatory syndrome (PRAAS)**

***Supplementary Material***

Jonas Johannes Papendorf^1^, Frédéric Ebstein^1,2^, Sara Alehashemi^3^, Daniela Gerent Petry Piotto^4^, Anna Kozlova^5^, Maria Teresa Terreri^4^ , Anna Shcherbina^5^ ,Andre Rastegar^3^, Marta Rodrigues^6^, Renan Pereira^7^, Sophia Park^3^, Bin Lin^3^, Kat Uss^3^, Sophie Möller^1^, Ana Flávia da Silva Pina^4^, Flavio Sztajnbok^6^, Sofia Torreggiani^3^, Julie Niemela^8^, Jennifer Stoddard^8^, Sergio Rosenzweig^8^, Andrew J. Oler^9^, Colton McNinch^9^, Marietta M. de Guzman^10^, Adriana Fonseca^6^, Nicole Micheloni^4^, Melissa Mariti Fraga^4^, Sandro Félix Perazzio^11^, Raphaela Goldbach-Mansky^3^, Adriana A. de Jesus^3,*^ and Elke Krüger^1,*^

^1^Institut für Medizinische Biochemie und Molekularbiologie (IMBM), Universitätsmedizin Greifswald, 17475 Greifswald, Germany

^2^Present address: Nantes Université, Inserm UMR 1087/CNRS UMR 6291, l’institut du thorax, 44000 Nantes, France

^3^Translational Autoinflammatory Diseases Section (TADS), Laboratory of Clinical Immunology and Microbiology, National Institute of Allergy and Infectious Diseases, National Institutes of Health, Bethesda, MD, United States

^4^Division of Pediatric Rheumatology, Department of Pediatrics, Universidade Federal de São Paulo (Unifesp), São Paulo, Brazil

^5^Department of Clinical Immunology, Center for Pediatric Hematology, Oncology, and Immunology, Moscow, USSR

^6^Division of Pediatric Rheumatology, Department of Pediatrics. Universidade Federal do Rio de Janeiro (UFRJ), Rio de Janeiro, Brazil

^7^Universidade Federal do Rio Grande do Sul, Porto Alegre, RS, Brazil

^8^Immunology Service, Department of Laboratory Medicine, Clinical Center, National Institutes of Health, Bethesda, MD, United States

^9^ Bioinformatics and Computational Biosciences Branch, Office of Cyber Infrastructure and Computational Biology, National Institute of Allergy and Infectious Diseases, National Institutes of Health, Bethesda, MD, USA

^10^Section of Pediatric Rheumatology, Texas Children's Hospital, Baylor College of Medicine, Houston, TX, USA

^11^Division of Rheumatology – Department of Medicine. Universidade Federal de São Paulo (Unifesp), Sao Paulo, Brazil

*Corresponding authors: [elke.krueger@uni-greifswald.de](mailto:elke.krueger@uni-greifswald.de) and [Adriana.almeidadejesus@nih.gov](mailto:Adriana.almeidadeejesus@nih.gov)

**Supplementary Table 2: Utilized Antibodies**

| **Primary Antibodies** | **Dilution** |
| --- | --- |
| LMP7/β5i (clone A7, sc-365699 Santa Cruz) | 1:10000 |
| V5 (clone 46-O705, Invitrogen) | 1:3000 |
| Alpha5/α5 (clone MCP196, Enzo Life Sciences) | 1:1000 |
| Rpt6 (clone p45-110, Enzo Life Science) | 1:6000 |
| β-actin (clone C4, sc-47778 Santa Cruz) | 1:2000 |
| HA (clone HA. 11, BioLeged) | 1:1000 |
| **Secondary Antibodies** | **Dilution** |
| Anti-mouse (#7076, Cells Signaling) | 1:10000 |

**Supplementary Table 3: Primers used for Plasmid Construct Creation**

| Gene | Primers for human genes (5’ → 3’) |
| --- | --- |
| *PSMB8* | Construct published (1) |
| *PSMA5* | KpnI_for GGGGGTACCATGTTTCTTACCCGGTCTGAGTACG  EcoRV_rev CCCCGATATCAATGTCCTTGATAACCTCTTCAAGT |
| *PSMC5* | NheI_for TTTTGCTAGCATGGCGCTTGACGGACCAGAGCAGA  KpnI_rev CCCCGGTACCCTCACTTCCATAATTTCTTGATGGAC |
| *PSMB10* | BamHI_for TTTGGATCCATGCTGAAGCCAGCCCTGGAGCCCC  XhoI_rev CCCCTCGAGAACTCCACCTCCATAGCCTGCACAGTT |

**Supplementary Table 4: Primers used for Mutagenesis**

| Mutagenesis | Primers for human genes (5’ → 3’) |
| --- | --- |
| *PSMB8* T75M | for CTTGAAGGCGAGCATGGTGGTGCCATGGG  rev CCCATGGCACCACCATGCTCGCCTTCAAG |
| *PSMB8* S118P | for GCTGCACAGCCAGGCATGGTGCCAAGC  rev GCTTGGCACCATGCCTGGCTGTGCAGC |
| *PSMB8* Q55* | for GGGGGATCCATGGCGCTACTAGATGTATGCGGAG  rev CCCCTCGAGTTGAAGAATTCTGTGGGCTGCATTCCC |
| *PSMA5* R168* | for CAGTGTGATGCTGAACAAAAACTCATCTCAGAAGAGGATC  rev GAGTTTTTGTTCAGCATCACACTGTACAAATGTCCCAGAT |
| *PSMC5* delExon 10 | for CCCCAATGAGGAGGGCGTGTGCACAGAAGCTGGCATGTA  rev CTCCTCATTGGGGCGTGGGAATTCAACTGTGCACACGCC |
| *PSMB10* F14del | for GAGGGGGCTTCTCCGAGAACTGCCAAAG  rev CTTTGGCAGTTCTCGGAGAAGCCCCCTC |
| *PSMB10* G167D | for CTTCACAGCCCTGGACTCTGGTCAGGACG  rev CGTCCTGACCAGAGTCCAGGGCTGTGAAG |

**Supplementary Table 5: Primers used for RNA RT-PCR**

| Primer Designation | Primer Sequence (5’ → 3’) |
| --- | --- |
| *RPLP0* | for CAATGGCAGCATCTACAACC  rev ACTCTTCCTTGGCTTCAACC |
| BGH_rev | rev GACAGTGGGAGTGGCACCTTCCAGGGTCAAGG |

**Clinical description patient 5**

**Patient 5** (2 novel *PSMB8* mutations previously reported)

A 13-year-old Caucasian girl born to healthy non-consanguineous parents, at 3 weeks of age, developed swollen and red fingertips, low grade fevers and episodic generalized erythematous maculopapular and nodular rashes involving the face, palms, and soles and bilateral swelling of the eyelids and lips that, up to age 3.5 years, were treated with topical medications. She developed iron deficiency anemia and received iron supplements. At the age of 4 years, she was started on hydroxychloroquine, but episodic skin flares every 3 to 4 months that would last for 2 weeks, and fever persisted, and she developed arthralgia, headache, and abdominal pain. The rashes were pruritic and accompanied by painful nodules on the face, palms, and soles. At the age of 5, persistent elevation of acute phase reactants led to a workup for malignancy including a bone marrow biopsy which were unrevealing and treatment with oral prednisone was initiated. At the age of 12 years, she was referred to the National Institutes of Health (NIH), her weight was 40.5kg (just below 50th percentile), and her height was 142.4 cm (10th percentile). She continued with frequent neutrophilic skin rashes, low-grade fevers, and had developed lipodystrophy. Other clinical features included myositis/fasciitis in both upper and lower extremities, bilateral basal ganglia calcifications and persistent headaches that worsened with episodic flares. The patient had a hemoglobin level of (Hb) 13 g/dl, hematocrit (Ht) 38.3%, white blood cell (WBC) count of 7,620 cells/mL, absolute neutrophil count (ANC) of 3,580 cells/mL, absolute lymphocyte count (ALC) of 3,580 cells/mL, and platelet count 129,000 cells/mL, erythrocyte sedimentation rate (ESR) 9 mm/hr, and C-reactive protein (CRP) 0.9 mg/L.

The disease flares were only responsive to methylprednisolone infusions or prednisone courses and for the last seven years, she was kept on relatively high doses of steroids (above 0.8 mg/kg/day) that could not be weaned without rebound flares. Several classes of steroid-sparing agents, including methotrexate, anti-TNF, IL-6 blocker, and IVIG were without success. At the age of 12 years, she was started on treatment with the JAK inhibitor, tofacitinib 5 mg twice a day, and still required short courses of prednisone during disease flares. Tofacitinib dose was increased to 10 mg twice a day (0.3 mg/kg/day) at the age of 16 years and her disease has been in remission for the past 3 years.


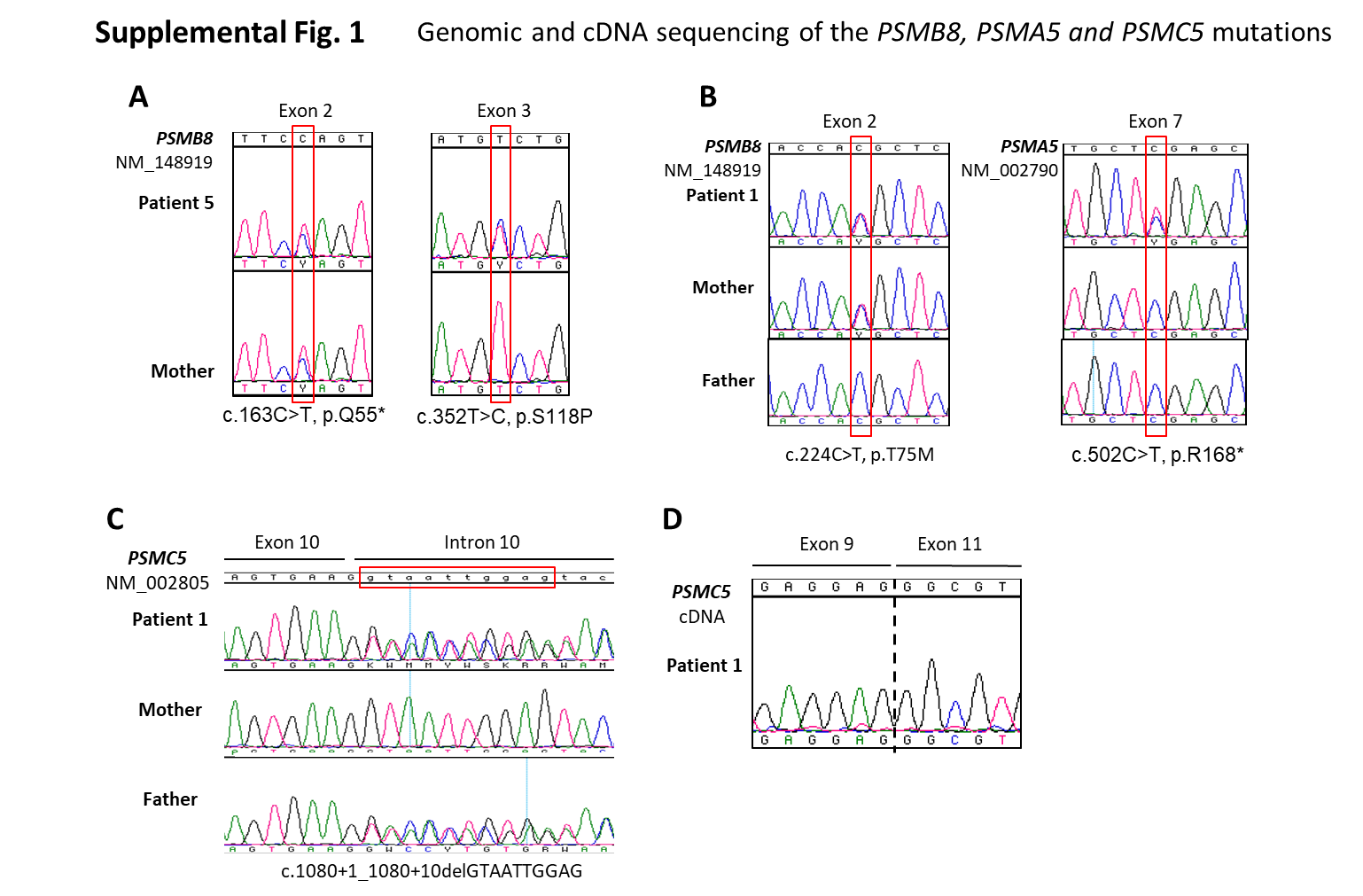


**Supplementary Figure 1.** Genomic and cDNA sequencing of the *PSMB8, PSMA5 and PSMC5* variants.(**A)** Sanger sequencing electropherogram depicting two novel compound heterozygous PSMB8 variants (c.163C>T, p.Q55* and c.352T>C, p.S118P) in Patient 5. (**B)** Sanger sequencing electropherogram of Patient 1 depicting a known CANDLE causing heterozygous *PSMB8* variant (c.224C>T, p.T75M) and a novel de novo heterozygous variant in PSMA5 (c.502C>T, p.R168*). (**C)** Sanger sequencing validation of *PSMC5* variant c.1080+1_1080+10delGTAATTGGAG in Patient 1. (**D)** cDNA sequencing for Patient 1 demonstrating skipping of exon 10 caused by the splicing disrupting variant c.1080+1_1080+10del.


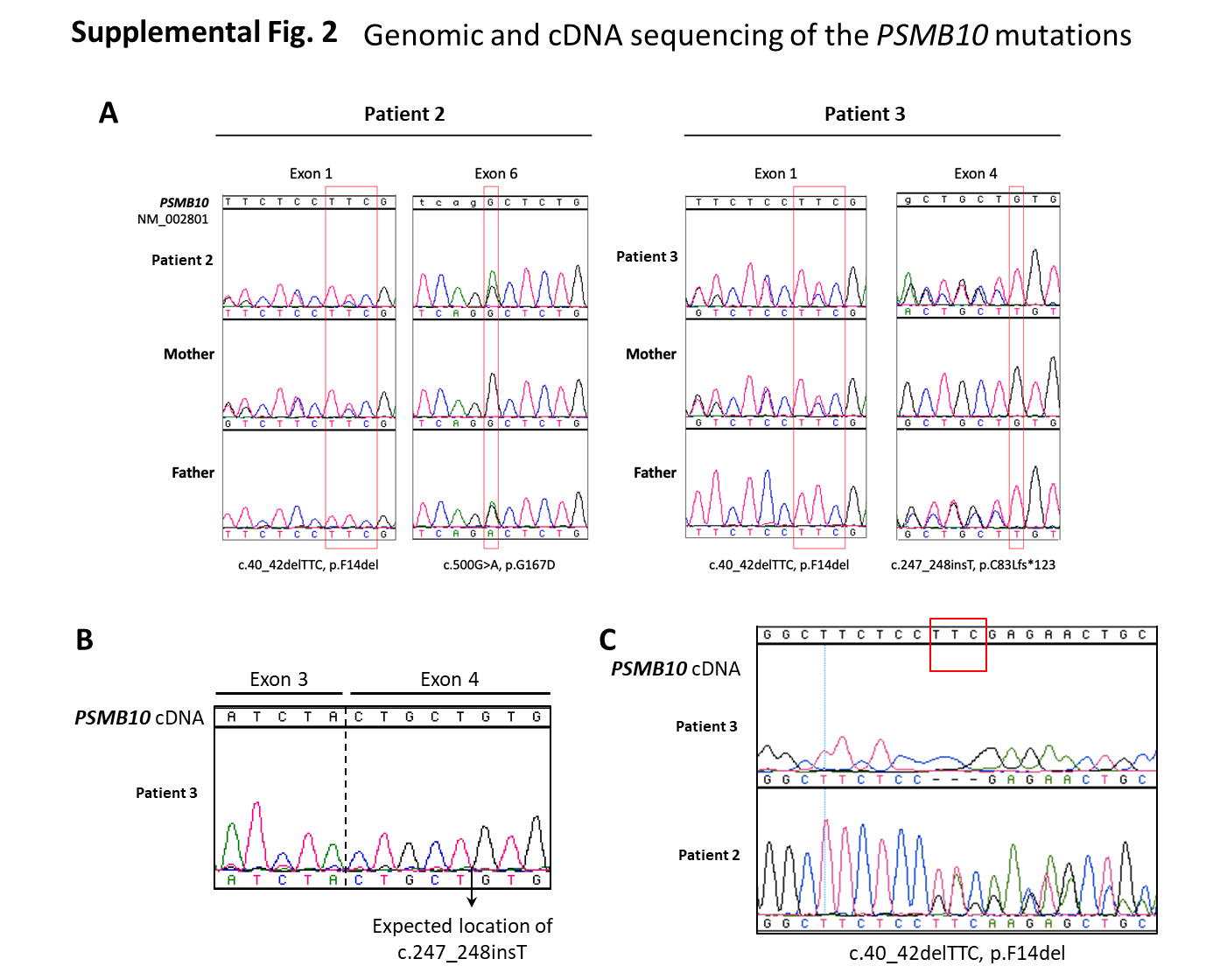


**Supplemental Figure 2.** **Genomic and cDNA sequencing of the *PSMB10* variants**. **(A)** Sanger sequencing validation of *PSMB10* variants in Patient 2 and Patient 3. **(B)** cDNA sequencing for Patient 3 showing the mutant (paternal) allele (c.247_248insT) is not expressed. Arrow shows the expected location of the insertion. **(C)** cDNA sequencing of *PSMB10* exon 1 for Patients 2 and 3 showing that in Patient 3 only mutant (maternal) allele (c.40_42del) is expressed. Wildtype (paternal) allele is not expressed in this area.


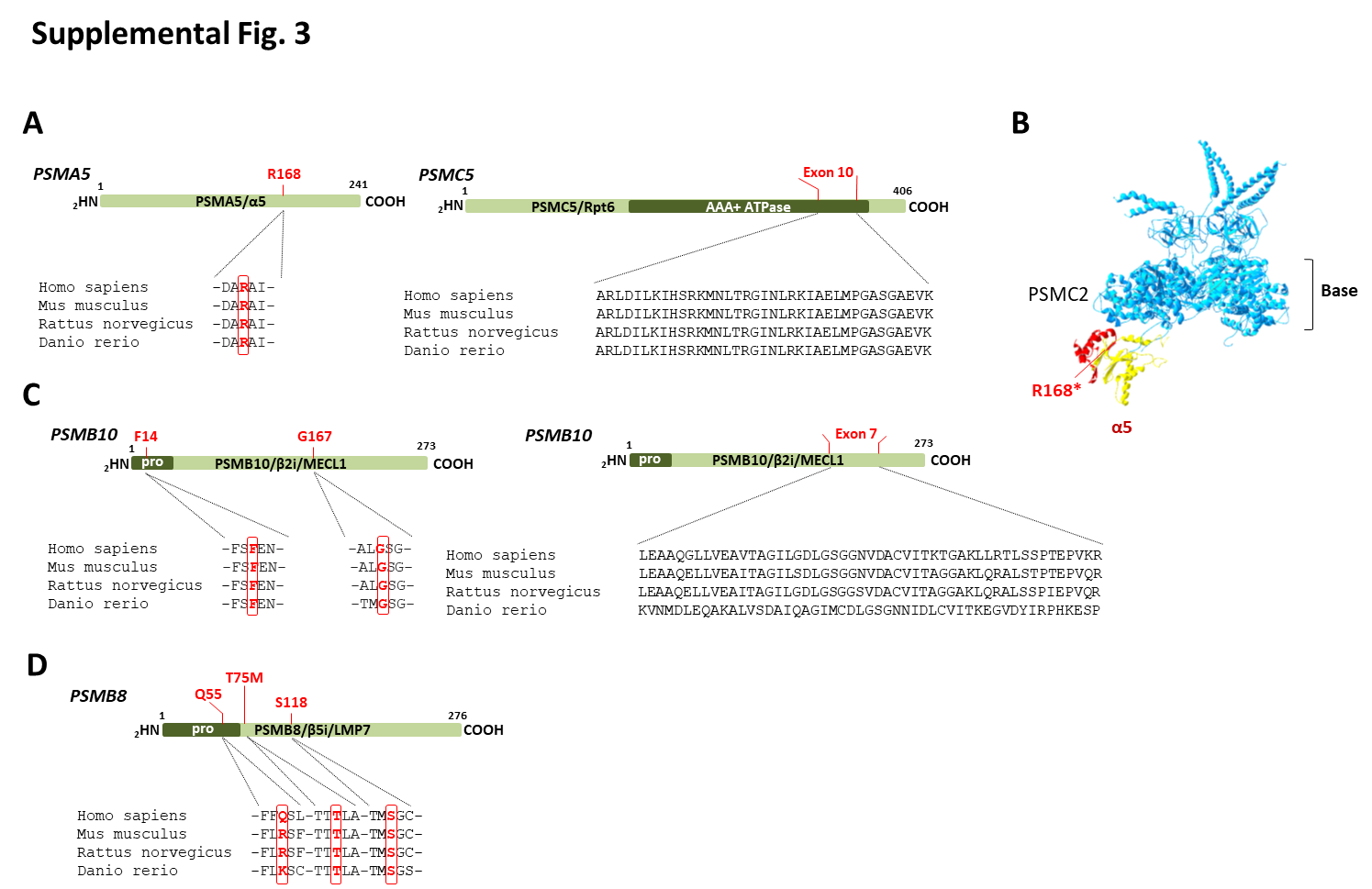


**Supplementary Figure 3. Protein localization and conservation of the seven identified alterations in PSMA5, PSMC5, PSMB10 and PSMB8.**The localization of the seven identified alterations in the **(A)** PSMA5 (α5), PSMC5 (Rpt6) and **(C)** PSMB10 (β2i/MECL1) or **(D)** PSMB8 (β5i/LMP7) proteins are indicated in red. Sequence alignment of the primary structures of protein regions subjected to variation across four eukaryotic organisms indicate high conservation of the affected residues. **(B)** *PSMA5*/ α5 lacks the C-terminal α-helices 4 and 5 which are predicted to bind *PSMC2*/Rpt1 ATPase of the 19S regulatory particle shown in the structure.

REFERENCES

1. Brehm A, Liu Y, Sheikh A, Marrero B, Omoyinmi E, Zhou Q, et al. Additive loss-of-function proteasome subunit mutations in CANDLE/PRAAS patients promote type I IFN production. *J Clin Invest* (2016) **126**:795. doi:10.1172/JCI86020
